# Perceived need and help-seeking for psychosocial support among health and social care professionals: a systematic review and meta-analysis

**DOI:** 10.1186/s12913-025-13214-6

**Published:** 2025-11-10

**Authors:** Oona Kuosmanen, Erika Jääskeläinen, Paulus Torkki, Kalle Seppälä, Eeva Tuunainen

**Affiliations:** 1 Faculty of Health Sciences, School of Medicine, Department of Emergency Medicine, University of Eastern Finland, Kuopio, Finland; 2 Research Unit of Population Health, University of Oulu, Oulu, Finland; 3 Department of psychiatry, Oulu University Hospital and Wellbeing Services County of Northern Ostrobothnia, Oulu, Finland; 4 Medical Research Center Oulu, Oulu University Hospital and University of Oulu, Oulu, Finland; 5 Department of Public Health, University of Helsinki, Helsinki, Finland; 6 Faculty of Medicine and Health Technology, School of Medicine, Department of Emergency Medicine, University of Tampere, Tampere, Finland

**Keywords:** Psychosocial support, Help-seeking, Need for support, Mental health, Health care professionals, Social care professionals, Systematic review

## Abstract

**Background:**

Health and social care professionals face multiple challenges, including a shrinking workforce, a rapidly aging population, and crises such as pandemics, which increase stress and the risk of mental health problems. Preventing these problems is essential to maintaining a stable workforce and ensuring the quality of care. This study investigates the proportion of health and social care professionals who have experienced a need for psychosocial support and those who have sought it. Additionally, the study evaluates key factors influencing help-seeking behavior.

**Methods:**

A systematic search of PubMed, PsycINFO, and CINAHL databases was conducted. Original studies with quantitative outcomes on the need for support and help-seeking among health and social care professionals were included, with no restrictions on the type of psychosocial support examined. The findings were categorized using the Quadruple Aim framework.

**Results:**

Sixteen studies were included in the systematic review, most of which focused on physicians (*n* = 11). The meta-analysis revealed that weighted average of 97.9% and unweighted average of 54.9% (range: 34–100%) of health and social care professionals perceived a need for psychosocial support (*n* = 6). Overall, 58.8% (range: 27–66%) sought some form of support (*n* = 4), while 30.2% (range: 7.2–72%) sought formal support (*n* = 12) and 67.4% (range: 58.2–78%) informal support (*n* = 6). The average prevalence of psychological distress and mental health problems among professionals was 55.3% (*n* = 12). Several studies also reported increased alcohol and substance use as well as barriers to help-seeking.

**Conclusion:**

This study underscores the substantial need for psychosocial support among health and social care professionals. Addressing this need is crucial not only for improving professionals’ mental health but also for sustaining the delivery of care. The gap between perceived need and actual help-seeking behavior must be addressed to develop effective and accessible support systems. By successfully bridging this gap, long-term sustainability of the health and social care workforce, and ultimately patient safety, can be enhanced.

**Supplementary Information:**

The online version contains supplementary material available at 10.1186/s12913-025-13214-6.

## Background

Healthcare professionals form the backbone of a functional health system, with their primary objective being to improve patient health. Consequently, safeguarding the mental health and well-being of healthcare workers is essential. Ensuring their well-being not only enhances the quality of care but also contributes to environmental sustainability within the healthcare sector while reducing the risk of work-related injuries and illnesses [28].

Several factors put a strain on healthcare professionals and challenge their ability to cope. Firstly, the rapidly aging population, coupled with the rise in multimorbidity and chronic conditions, has led to an increased demand for healthcare professionals [29]. Additionally, demographic shifts, particularly in Western countries, are expected to result in a shrinking healthcare workforce. Many healthcare systems are already struggling to meet the rising demand for care, as workforce shortages become an increasing concern.

However, an insufficient workforce is not the sole factor contributing to strain or possible deterioration of occupational well-being. The inherently stressful nature of healthcare work, places professionals at increased risk of mental health problems. Emergency medicine professionals, in particular, face high levels of emotional, physical, and psychological stress during each shift, significantly increasing their risk of job-related burnout, depression, and suicide [30].

Similarly, social care professionals also face substantial workloads and role conflicts, which contribute to job-related stress and may even influence their attitudes toward clients [31]. According to a study by Tham [32], 48% of social workers intended to leave their jobs, with the most commonly cited reason being a lack of human resource orientation within their organizations. This often manifests as a perception that management is uninterested in employees’ well-being. Such findings highlight the critical role of organizational factors in shaping workplace well-being. Furthermore, studies have demonstrated that the risk of work disability due to job-related stress is equally high among social care professionals as it is among other healthcare professionals [33].

The COVID-19 pandemic has further exacerbated psychological distress and mental health problems among healthcare workers, as professionals were required to perform their duties under extreme stress. The pandemic itself was identified as an independent risk factor for deteriorating mental health [34]. A report by the Qatar Foundation, WISH, and the WHO revealed that during the pandemic, 23–46% of healthcare workers reported symptoms of anxiety, while 20–37% experienced symptoms of depression [35]. Burnout was even more prevalent, affecting 41–52% of professionals during the pandemic. As pandemics and other global crises become more frequent, new strategies must be developed to mitigate their psychological impact on healthcare professionals [36].

Supporting the mental health of healthcare professionals is also a matter of patient safety. According to Hall et al. [37], poor well-being and burnout among healthcare workers are associated with negative patient safety outcomes, including an increased likelihood of medical errors. Additionally, gender disparities must be considered, as women constitute 89% of nurses, 98% of midwives, and 49% of doctors [38]. From an economic perspective, investing in psychosocial support yields significant returns: ”Every US$ 1 invested in scaling up treatment for common mental illnesses such as depression and anxiety leads to a return of US$ 4 in better health and ability to work.” [39]. Conversely, failing to address mental health problems is projected to result in an annual global economic loss of approximately one trillion US dollars.

Given the increasing challenges faced by healthcare and social care professionals, it is essential to assess the impact of mental health problems and adverse events on their well-being. Mental health problems left untreated may escalate into diagnosable mental disorders and lead to work incapacity. Psychosocial support is recognized as a widely accepted first-line intervention for psychological distress and mental health issues. Although research on these topics is emerging, to our knowledge, no previous systematic review has specifically examined the perceived need for psychosocial support and help-seeking behavior among health and social care professionals.

### Study objectives

To effectively promote the mental health and well-being of professionals, it is important to understand their need for psychosocial support. It is also crucial to determine the sources from which professionals seek support and the circumstances in which they require it. Equally important is identifying the reasons why professionals may not seek support even when they need it. By recognizing both the demand for psychosocial support and the barriers to help-seeking, employers may be better equipped to implement targeted and accessible support systems for their workforce.

The objectives of this study were as follows:

1. To determine the proportion of health and social care professionals who have experienced a need for psychosocial support. Additionally, we aimed to assess the range of psychological distress and mental health problems experienced by these professionals.
2. To determine the proportion of health and social care professionals who have sought psychosocial support. We define help-seeking behaviour as an active process initiated by the individual, such as making an appointment with a mental health professional or reaching out to a colleague for personal support.

Furthermore, we aimed to examine whether any background factors influenced help-seeking behavior or acted as barriers to seeking support. These factors were analyzed based on the selected studies.

### Hypothesis

We hypothesized that there is a significant need for psychosocial support among health and social care professionals and that a considerable proportion of those in need do not seek help. We anticipated that various factors, such as limited availability of support services or difficulties in accessing them, may contribute to the observed gap between the need for and the utilization of psychosocial support.

## Methods

This systematic review followed the PRISMA guidelines [40]. The PRISMA checklist is available in the Supplementary Data file.

### Literature search

A comprehensive literature search was conducted on February 10, 2024, using three electronic databases: PubMed, PsycINFO, and CINAHL. The search strategy was developed in consultation with an informatician and included three primary components:

1. **Psychological distress and mental health problems**, including terms such as *stress*, *burnout*, and *empathic distress*.
2. **Health and social care professionals**, with terms such as *social care work*, *nurse*, and *hospital profession*.
3. **Need for support and help-seeking**, using terms such as *need*, *support*, *help*, and *perceived*.

To refine the search results, the additional term **[Title]** was used to ensure the retrieval of studies with a clear focus on these topics. The final search phrase was as follows:

(“mental health*” OR psychological* OR stress OR burnout OR burn-out OR “burn out” OR fatigue OR distress OR anxiety OR insomnia OR depression OR sleep* OR “compassion fatigue” OR “empathic distress”)

AND.

(“social care work*” OR “social care profession*” OR “health care work*” OR “health care profession*” OR “hospital work*” OR “hospital profession*” OR “medical work*” OR “medical profession*” OR nurse* OR doctor* OR physician*)

AND.

(need* OR support OR help* OR perceived)

Additionally, a manual search of the reference lists of included articles was performed to identify any relevant studies not retrieved through the database search.

### Inclusion and exclusion criteria

Studies were included if they met the following criteria:

1. Reported quantitative outcomes related to either the perceived need for psychosocial support or help-seeking behavior.
2. Focused on a study population of health and/or social care professionals.
3. Were published in English, Finnish, Swedish, German, or French.
4. Were peer-reviewed journal articles.

There were no restrictions on publication year or sample size. Review articles and case reports were excluded.

Only studies reporting quantitative outcomes were included. Eligible studies were required to use a clear and explicit measure of the need for support or help-seeking, such as:

- *“Have you ever sought help from or contacted a mental health professional?”*
- *“How often have you felt the need to discuss your stress/problems with a mental health professional in the past month?”*

Studies reporting only the receipt of support (rather than the perceived need for or seeking support) were excluded, except in cases where receiving and seeking support were used synonymously (e.g., studies using the question *“If you experienced mental health problems in the past year*,* did you seek/receive help for them?“*).

Studies using the following formulations were also excluded, as they did not clearly indicate whether professionals really perceived need for support or sought it:

- *“I feel like my superior/friend/colleague supports me.”*
- *“I would seek help if…"*.

Additionally, studies utilizing the following questionnaires were excluded, as these instruments did not provide information directly relevant to our eligibility criteria:

- Social Support Rating Scale (SSRS).
- Perceived Social Support Scale (PSS).
- Multidimensional Perceived Social Support Survey (MPSS).
- Perceived Organizational Support (POS).
- Job Content Questionnaire (JCQ).

All types of psychosocial support were considered, including support from professionals, friends, family, colleagues, and supervisors.

### Screening and data extraction

The abstracts of all search results were independently screened by two researchers (OK and EJ/PT/ET) using the Covidence tool [41]. Any disagreements were resolved through discussion among the authors. Full-text reviews were conducted by one reviewer (OK), and in cases of uncertainty, a second author reassessed the article. Following the full-text review, a secondary evaluation of the selected studies was performed by another author (EJ).

### Quality assessment

The JBI Critical Appraisal Checklist [42, 43] was used to assess the quality of the included studies. One reviewer (OK) conducted the quality assessment, and the quality ratings are presented in the Supplementary Data file.

### Data analysis

Following the full-text review, the following variables were extracted and independently analyzed (by OK):

1. Basic study information: Title, author, publication year, country, and study design.
2. Sample characteristics: Sample size, gender distribution, age, profession, and work-related characteristics.
3. Primary outcomes: Perceived need for psychosocial support and help-seeking behavior, categorized as:

. Overall help-seeking (cases where the provider of support was either unclear or impossible to determine).

. Formal help-seeking (support sought from professionals or trained personnel).

. Informal help-seeking (support sought from friends, family, colleagues, supervisors or social networks).

Additional findings, including:

- Prevalence of mental health problems or psychological distress in the sample.
- Sources of help-seeking.
- Alcohol/substance use.
- Barriers to help-seeking.
- Other relevant findings.

A descriptive analysis was performed for these factors. Additionally, a meta-analysis was conducted when at least three original studies reported comparable outcomes.

In two of the three studies examining both the need for support and help-seeking, the help-seeking analysis was conducted exclusively on participants who had also expressed a need for support. In these instances, we assumed that individuals who did not report needing support also did not seek it. Consequently, these two studies were included in the meta-analysis for help-seeking behavior.

We first grouped the studies by outcomes and then assessed the average and 95% confidence intervals for proportion of persons perceiving need for support and proportion of persons seeking help in each group. In meta-analysis, we used both weighted and unweighted averages as there were not so many included studies and the size of study populations differed a lot. The weighted average gives an accurate estimate of the combined effect of the results as the unweighted average gives an overview without statistical weighting helping to detect if there are any conflicting results. IBM SPSS Statistics 29.0.2.0 was used for the calculations.

## Results

**Fig. 1:**
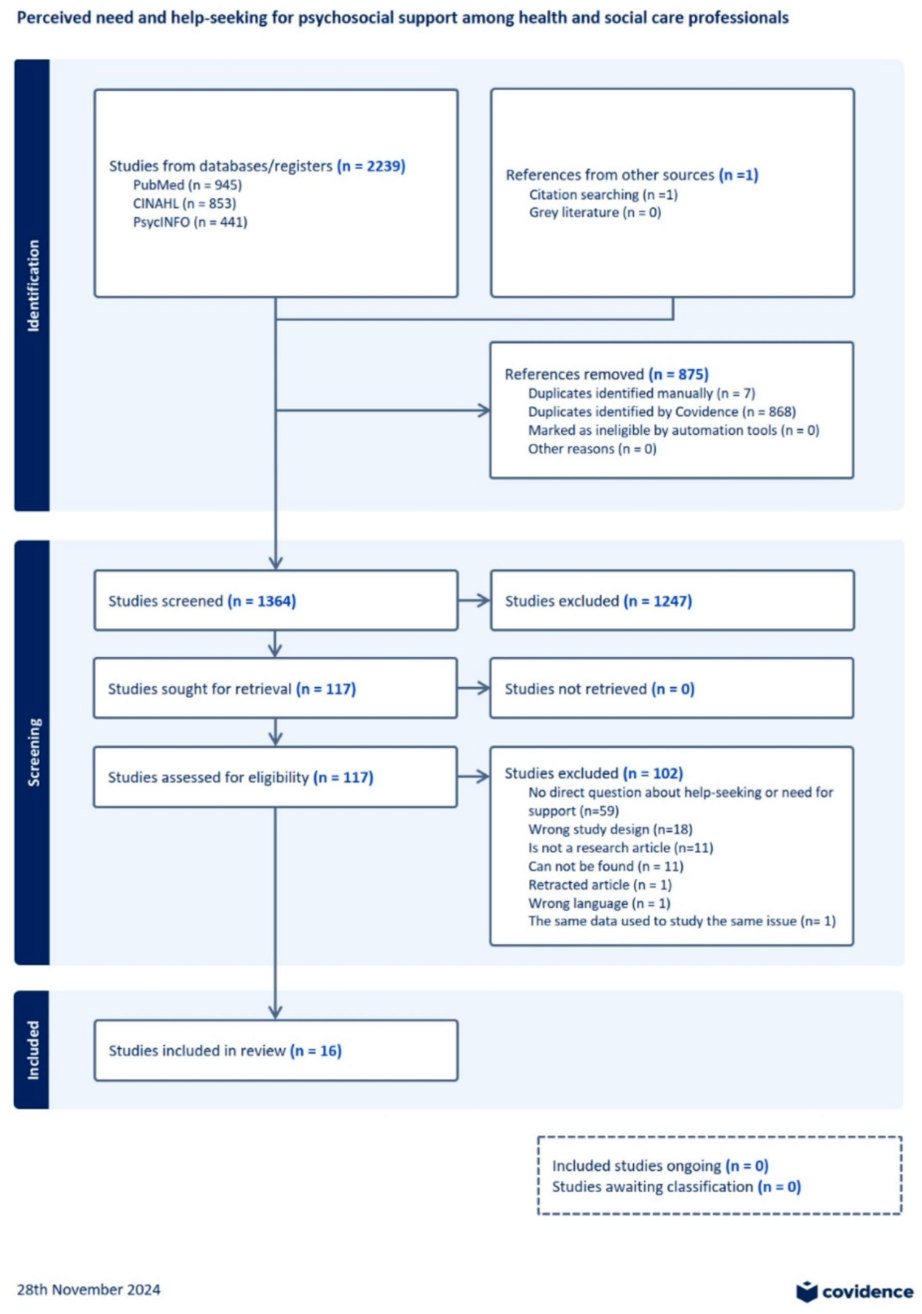
PRISMA flow diagram from Covidence tool

The database search yielded a total of 2,239 results, including 945 records from PubMed, 853 from CINAHL, and 441 from PsycINFO (Fig. 1). The Covidence tool identified and removed 868 duplicate entries, while an additional 7 duplicates were manually removed. Following the removal of duplicates, 1,364 studies remained for title and abstract screening. Based on this initial evaluation, 117 publications were selected for full-text review. Of these, 15 studies met the inclusion criteria. An additional publication, identified through reference screening of the selected studies, also met the inclusion criteria. Consequently, a total of 16 studies were included in the final review.

### Characteristics of the sample

The majority of the studies (9 out of 16) were published in 2020 or later. The samples were geographically diverse, with no single country being predominant. Most studies employed a cross-sectional design (*n* = 15), while one study utilized a longitudinal design. Two studies employed mixed methods; however, only the quantitative results were included in this review. Sample sizes ranged from 27 to 51,406 participants. In ten studies, the sample size was fewer than 1,000, while six studies included more than 1,000 participants. The proportion of female participants across the studies averaged 56.3% (range: 13.3–100%). Two studies did not report gender distribution, and in one study, it was difficult to assess overall gender distribution across all age groups.

The study populations comprised physicians in 11 studies, nurses in one study, social care workers in one study, healthcare workers in one study, and both healthcare and social care workers in two studies.

Most of the studies focused exclusively on help-seeking behavior (*n* = 10), while three studies examined the need for support, and an additional three studies explored both outcomes (Table 1). These outcomes were measured using various self-reported questionnaires. Among the studies investigating the need for support, three focused solely on formal support or professional help, while the provider of support was unclear in the other three. Of the ten help-seeking studies, six investigated both formal and informal support. Another six reported only formal support or professional help-seeking, and in one study, the provider of support was not specified.

**Table 1:** Background information and results of the included studies

Study	Authors, Publication year, Country	Population	Sample size	Gender	Need/seeking for support (%), help-seeking source	The proportion of sample reporting mental health problems or psychological distress (%)
1	W. Zhang et al. 2023, China	nurses	51 406		100% needed support, formal (psychological counseling)	95.9% reported some form of occupational stress
2	R. Tyssen et al. 2004, Norway	junior doctors	327	54% women	34% needed support, 64% sought support (formal)	
3	M. Schiff et al. 2018, Israel	foster care social workers	82	100% women	21.7% needed support (unclear)	26.7% reported symptoms of partial PTSD
4	D.A. Castelli Dransart et al. 2020, Switzerland	health and social care workers	704	65.6% women	39.1% needed support, 39.3% sought support (unclear/overall)	
5	S. Grover et al. 2019, India	doctors (medical residents)	442	30.3% women	73.1% needed support, 12.9% sought support (formal)	91.2% reported moderate or higher level of stress
6	PTL Nguyen et al. 2021, Vietnam	medical and non-medical health care workers	761	58.2% women	61.6% needed support (unclear)	34.3% had psychological stress symptoms
7	R. Styra et al. 2022, Canada	health and social care workers	3852	84.2% women	8.2% sought formal support 77.3% sought informal support	
8	C. Wijeratne et al. 2021, Australia	doctors	4067 *	hard to evaluate	66% overall sought support 72% sought formal and 64% informal support	40.3% had a lifetime history of depression *
9	M. Ramzi et al. 2020, Australia	doctors	4154 *	55.7% women	64.9% overall sought support 60% sought formal and 58.2% informal support	33.9% had ever felt seriously depressed *
10	M. B. King et al. 1992, UK	doctors	133 *	31.9% women *	20.3% sought formal and 72.9% informal support	68% have ever had periods of moderate or severe emotional distress *
11	K. Gupta et al. 2022, USA	doctors	1292 *		27% overall sought support, 13% sought formal and 78% informal support	56% of all respondents reported at least some burnout 76% reported involvement in an adverse event *
12	A. Kruper et al. 2020, USA	ob-gyn doctors	27	88% women	12% sought formal and 69% informal support	90% reported being involved in an adverse medical event 81% reported anxiety, 62% quilt and 58% disrupted sleep
13	A. Fridner et al. 2012, Italy, Sweden	university hospital doctors	516 *	46.1% women	21.1% sought formal support	42.7% showed one or more indices of current psychological ill-health *
14	N. Jones et al. 2018, UK	military doctors	678	27.8% women	35.6% sought formal support	46.1% reported stress, emotional or mental health problems occurring in the last three years
15	D. Guizar-Sanchez et al. 2022, Mexico	family doctors	263 *	77.0% women *	8% sought formal support	47.9% reported mental health problems *
16	T. D. Shanafelt et al. 2011, USA	doctors (surgeons)	7905	13.3% women	7.2% sought formal support	6.4% reported suicidal ideation

* **Study 8**: The original sample comprised 10,083 participants, with help-seeking analysis limited to 4,067 doctors with a lifetime history of depression. Mental health problems were calculated based on the full study population (4,067/10,083)

**Study 9**: Among 12,252 participants, help-seeking was analyzed for 4,154 doctors who reported ever feeling seriously depressed, even without a formal diagnosis. Mental health problems were calculated from the full sample (4,154/12,252)

**Study 10**: The study included 210 participants, with help-seeking analysis conducted among 133 doctors who reported past emotional distress and completed the full questionnaire. Gender data were based on the original sample (210), and mental health problems were calculated from the full population (141/210)

**Study 11**: This study had 1,808 participants, with help-seeking analysis focusing on 1,292 doctors who experienced emotional impact from an adverse event. Burnout was reported in 1,006 participants, and 1,372 were involved in an adverse event (out of 1,808)

**Study 13**: The original sample included 1,208 participants. The study analyzed help-seeking among 516 physicians exhibiting one or more indicators of current psychological ill-health. Mental health problems were calculated from the original sample (516/1,208)

**Study 15**: Of 566 doctors, help-seeking analysis was conducted among 263 physicians responsible for a patient who died under their care. Gender data were reported as a percentage of the full sample (566), and mental health problems were calculated from the original population (271/566)

### Meta-analysis

Meta-analysis was performed for both outcomes, the need for support (*n* = 6) and help-seeking behavior (*n* = 13).

#### Need for support

**Fig. 2:**
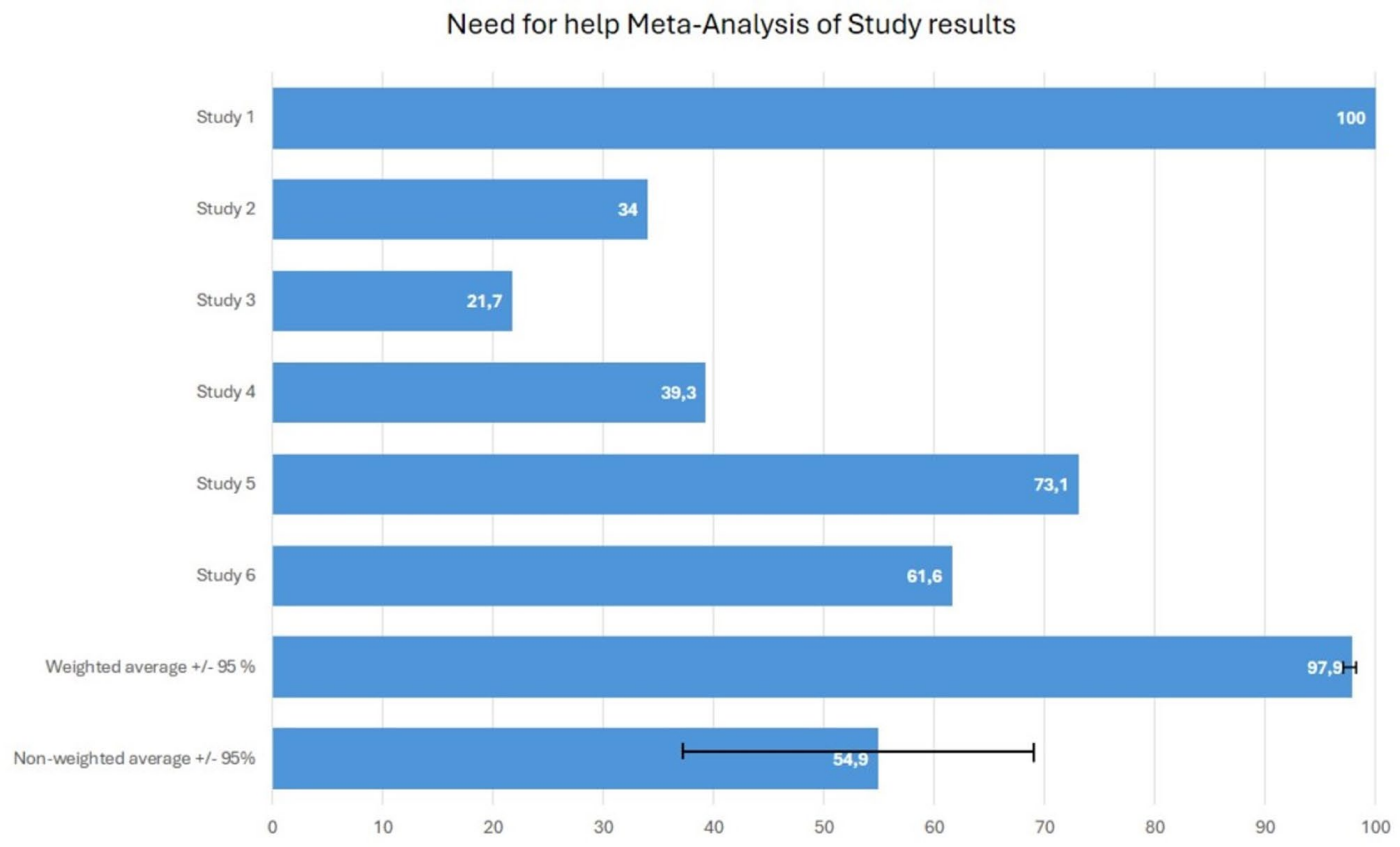
The proportion of individuals reporting need for psychosocial support in included studies (*n* = 6). Study 1: This study had a large sample size (51,406), with all participants reporting at least some level of need for support, measured using a Likert scale. It had the greatest impact on the weighted average, which ultimately approached 100%. The study focused on the need for support from formal sources. Study 2: This study examined the need for support from formal sources among professionals who had experienced mental health problems within the past year. Study 3: In this study, the source of needed support was not specified. The need for support was assessed among professionals who had been exposed to armed conflict. Study 4: Similar to Study 3, the source of needed support was unclear. The need for support was evaluated among professionals whose patients had recently died. Study 5: This study assessed the need for support from formal sources. Study 6: The study investigated various Covid-19-related demands for mental health support

The meta-analysis indicated that a weighted average of 97.9% and unweighted average of 54.9% (range: 34–100%, 95% CI 37.3% − 69.3%) of health and social care workers reported some form of need for psychosocial support (*n* = 6). In three of these studies, the need was specifically for formal support, while in the remaining three studies, the preferred source of support was not specified (Fig. 2).

#### Help-seeking

The meta-analysis revealed that a weighted average of 58.8% and unweighted average of 49.3% (range: 27–66%) of health and social care workers sought some form of support, although the specific providers of support were not identified (*n* = 4).

We further analyzed formal and informal help-seeking. The weighted average for seeking formal support was 30.2% and unweighted average was 27.9% (range: 7.2–72%, 95% CI 15.0%-42.8%) among health and social care workers (*n* = 12). In contrast, a weighted average of 67.4% and unweighted average of 69.9% (range: 58.2–78%, 95% CI 63.8%-76.2%) of workers sought informal support (*n* = 6).

**Fig. 3:**
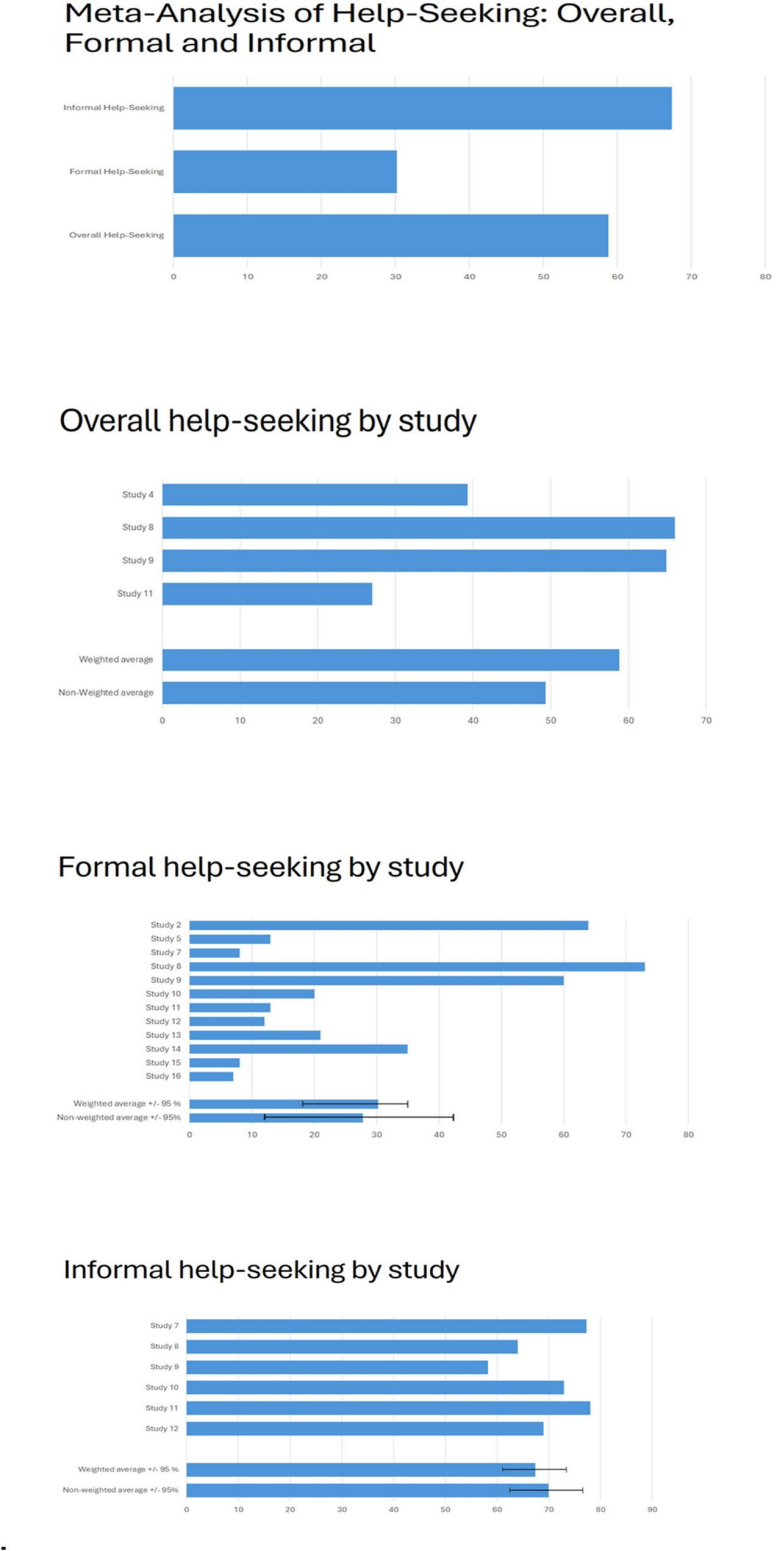
**a** A summary of the proportions of individuals reporting overall, formal and informal help-seeking. **b** The proportion of individuals reporting overall help-seeking (*n* = 4). **c** The proportion of individuals reporting formal help-seeking (*n* = 12). **d** The proportion of individuals reporting informal help-seeking (*n* = 6). Study 2. * Help-seeking was assessed exclusively among professionals who had experienced mental health problems within the past year. Study 4. * Help-seeking was evaluated among professionals whose patients had recently died. The source of support sought was not specified. Study 5: -. Study 7. **. Study 8. In this study, help-seeking results were stratified by three different age groups. We computed an overall result by combining data from these categories. For formal and informal help-seeking, we selected the values from the age group (younger practitioners) and support providers (formal: general practitioner, informal: friends) that had the highest reported rates. Help-seeking was evaluated among professionals with a lifetime history of depression. Study 9. Help-seeking was assessed among professionals who had ever experienced serious depression. Study 10. The focus was on professionals who had experienced emotional distress. Study 11: ** Help-seeking was assessed in professionals affected by the emotional impact of an adverse event. Study 12: ** The study evaluated help-seeking among professionals who had experienced secondary traumatic stress. Study 13: -. Study 14: -. Study 15: Help-seeking was assessed only among professionals who were attending physicians of a patient who died. Study 16: -. * Help-seeking was assessed only among professionals who had also expressed a need for support. ** Multiple values for informal and formal help-seeking were reported based on the provider of support (e.g.,* friends*,* colleagues*,* partner*,* or family). For the meta-analysis*,* we selected the highest reported values*

Specifically, 6 out of 13 studies on help-seeking behavior examined professionals who were already experiencing psychological distress or mental health problems, and an additional 2 studies focused on professionals whose patients had recently died (Fig. 3).

### Psychological distress and mental health problems among health and social care professionals

All included studies (*n* = 16) examined psychological distress or mental health problems among health and social care professionals. The proportion of participants reporting mental health problems or psychological distress could be assessed in 12 studies. Across these studies, the average prevalence of mental health problems was 55.3% (range: 26.7–95.9%), encompassing conditions such as occupational stress, anxiety, depression, and PTSD symptoms. Variability in prevalence rates was primarily due to differences in study scope and the types of mental health problems assessed. For example, in the study by Zhang et al. (Study 1 [Zhang et al., 2023]), 95.9% of nurses reported some level of occupational stress, whereas in the study by Schiff et al. (Study 3 [Schiff et al., 2018]), 26.7% of social care workers exhibited symptoms of PTSD.

Additionally, two studies reported suicidal ideation among healthcare professionals. In the study by Shanafelt et al. (Study 16 [Shanafelt et al., 2011]), 6.4% of surgeons reported experiencing suicidal ideation. In the study by Fridner et al. (Study 13 [Fridner et al., 2012]), 30% of professionals currently experiencing psychological ill-health reported suicidal thoughts. Twelve of the included studies assessed help-seeking behavior or the need for support in populations that had already experienced psychological distress, such as depression, burnout, or trauma from adverse events (e.g., patient suicide, medical error, or other traumatic incidents).

### Alcohol and substance use

Alcohol and substance use were reported in four studies (25%). Six studies (37.5%) assessed stigma or other barriers to help-seeking.

Styra et al. (Study 7 [Styra et al., 2022]) reported that 26.4% of allied health workers (including social workers), 27.5% of nurses, and 25.0% of physicians had initiated or increased alcohol consumption, though this result was not statistically significant (*p* = 0.793). Additionally, 10.9% of allied health workers, 19.3% of nurses, and 8.5% of physicians reported using hypnotics to aid sleep (*p* < 0.001). Alcohol use and perceived lack of support were associated with moderate to severe anxiety, PTSD symptoms, and depression.

King et al. (Study 10 [King et al., 1992]) found that 18 out of 133 doctors (13.5%) had increased their alcohol intake to cope with emotional distress. Similarly, Grover et al. (Study 5 [Grover et al., 2019]) reported that 25.5% of doctors started or increased substance use due to work-related stress, and 13.2% admitted to self-medicating with drugs for psychiatric disorders. Jones et al. (Study 14 [Jones et al., 2018]) found that 3.3% of military doctors reported having an alcohol problem within the past three years, and 13.6% had sought help for alcohol use.

### Stigma and barriers to help-seeking

Barriers to help-seeking were frequently reported across studies. Only the medical profession is mentioned because the available data on stigma and barriers to help-seeking came exclusively from studies involving physicians.

Wijeratne et al. (Study 8 [Wijeratne at al., 2021]) found that fear of lack of confidentiality (41–59%) and possible embarrassment (26–43%) were the most commonly cited barriers to seeking support in all age groups. Stigma was reported as a barrier by 15–30% of participants. A significant proportion of doctors believed that experiencing anxiety or depression was a sign of personal weakness (ranging from 31% in older doctors to 51% in younger doctors). Many also found the idea of being a patient embarrassing (48–63%). Ramzi et al. (Study 9 [Ramzi et al., 2021]) reported that fear of lack of confidentiality (61.9%) was the most common barrier to help-seeking, followed by embarrassment (44.8%) and stigmatizing attitudes (32.9%). King et al. (Study 10) found that 10 out of 133 doctors (7.5%) were embarrassed to seek help, and 7 doctors (5.3%) were concerned about confidentiality.

In the study by Grover et al. (Study 5), 58.1% of doctors believed that people were reluctant to seek help within the institution due to fear of being labeled as weak or unable to handle pressure. Additionally, 54.6% identified stigma as a barrier, and 36.3% cited concerns about confidentiality.

Jones et al. (Study 14) found that 67.3% of military doctors were concerned about stigmatization and did not want a mental health problem to be on their medical records. The most common perceived barrier to care was difficulty obtaining time off work for treatment (45.8%). A majority of participants (59.6%) expressed a preference for solving problems independently, while 65.5% reported that seeking support would make them feel inadequate. Furthermore, 48.3% found help-seeking too embarrassing, and 23.7% were worried about confidentiality.

Finally, Shanafelt et al. (Study 16) reported that 38.8% of surgeons would be reluctant to seek help for mental health problems due to concerns that doing so might negatively impact their license to practice medicine.

It was not possible to evaluate the costs associated with mental health problems or the consequences of not seeking help, as the majority of included studies did not provide relevant data. Only one study (Study 10) reported information regarding time off work. In this study, 12 out of 133 doctors (9%) indicated that they had taken time off work due to emotional distress. None of the included studies provided estimates of the financial costs associated with mental health outcomes or their impact on workplace productivity.

## Discussion

### Summary of findings

To our knowledge, this is the first systematic review synthesizing the extent of perceived need for psychosocial support and help-seeking behavior among health and social care professionals. Our primary findings indicate that up to 97.9% (un-weighted average of 54.9%) of health and social care professionals reported some form of need for psychosocial support, while 30.2% sought formal support and 67.4% sought informal support. In cases where the source of help-seeking was unclear or could not be determined, the overall help-seeking rate was 58.8%.

Our results demonstrate a significant demand for psychosocial support. Furthermore, the disparity between formal and informal help-seeking is noteworthy. A large proportion of included studies assessed help-seeking among individuals who were already experiencing depression or other mental health challenges. Given this context, the help-seeking rates remain relatively low. The gap between the perceived need for support and the actual help-seeking behavior suggests that, for various reasons, many health and social care professionals do not seek the support they require.

### Barriers to help-seeking and alternative coping mechanisms

This review identified several barriers to help-seeking. In six studies, embarrassment, concerns about confidentiality and privacy, and stigma were the most frequently reported obstacles. These findings suggest that negative attitudes toward seeking support are deeply entrenched, particularly within the medical profession.

Additionally, four studies reported that 3.3–27.5% of health and social care professionals had problems with alcohol use or had increased their consumption of alcohol or other substances. This finding suggests that a significant proportion of professionals may use substances as a coping mechanism for their mental health challenges instead of seeking appropriate support. Such self-medicating behavior may further contribute to the gap between the need for and the use of psychosocial support services.

### Comparison with previous research

Previous systematic reviews on this topic have primarily focused on the prevalence of psychological distress or mental health problems and overall mental health among professionals [1, 2, 37]. Research on the mental health and well-being of health and social care professionals has been particularly prominent in the context of the COVID-19 pandemic [3, 4]. Additionally, systematic reviews have examined barriers to help-seeking and interventions for support [5–8]. The role of social support, including perceptions of support received from supervisors, colleagues, friends, and family, is also well-documented in the literature [9–11]. However, few studies have specifically investigated the perceived levels of experienced need for support and the help-seeking behaviors of health and social care professionals.

This systematic review contributes valuable quantitative data on the perceived need for psychosocial support interventions and their utilization among health and social care professionals.

### Cost considerations

We also aimed to evaluate the financial implications of mental health problems and psychological distress and the consequences of not seeking support. However, only one included study provided data on time taken off work due to distress, and none estimated the associated costs. As a result, assessing the direct economic impact of mental health problems and psychological distress among health and social care professionals proved challenging. Further research is needed on this issue.

## Strengths and limitations

This study has several strengths. We included all health and social care professionals in the search strategy, ensuring a broad scope of analysis. A professional informatician was consulted to optimize the search strategy, and the final search terms were carefully reviewed and approved. We included all forms of needed and sought support, allowing for a comprehensive evaluation of the impact of different support sources on help-seeking behavior. Furthermore, we imposed no restrictions on publication year or study sample size.

In addition to the meta-analysis on the need for and seeking of support, we assessed background factors, including the prevalence of psychological distress and mental health problems, barriers to help-seeking, and substance use, that may explain some of our key findings. Presenting systematic quantitative data on the need for and seeking of support may provide valuable insights for future research. The findings of this study highlight the importance of addressing these issues and may serve as a foundation for developing new models and interventions to support the well-being of health and social care professionals.

Nonetheless, this study has some limitations. We included only articles published in English, German, French, Finnish, and Swedish, with all ultimately included studies being written in English. This language restriction may have resulted in the omission of relevant findings. Additionally, our inclusion criteria for the need for and seeking of support were relatively strict, as we did not include studies that only reported on received support or perceived social support from colleagues or supervisors. Given the complexity and multidisciplinary nature of this research field, there is also a possibility that some relevant studies were not identified. Furthermore, the relatively small number of included studies may affect the generalizability and reliability of the results. Also, the sample size varied across the studies. This heterogeneity affects the results of the meta-analysis especially considering the need for support. Additionally, as we did not limit inclusion based on the specific psychological symptoms for which professionals sought or needed support, this variability may have affected our findings. The impact of heterogeneity is particularly relevant to our results on help-seeking behaviour, given the wide range of informal support sources included, such as support from friends and family, as well as institutionally based support. Furthermore, only a small number of studies disaggregated findings by gender, ethnicity, or career stage, which limits the granularity and interpretive depth of our analysis.

Given the variability in terminology and reporting practices across the included studies, we recommend the development of a standardized framework for reporting help-seeking behaviour and perceived need for psychosocial support. Below we provide a framework with recommendations for future primary studies (Table 2).

**Table 2:** A standardized framework for reporting help-seeking behaviour and perceived need for psychosocial support

Component	Recommendation	Example / Note
1. Assessment of perceived need and help-seeking for psychosocial support	Use clearly phrased binary (yes/no) items to assess both perceived need and help-seeking behaviour.	Example items: “I have felt that I needed psychosocial support for my stress” (Yes/No) “I have sought psychosocial support for my stress” (Yes/No)
2. Relationship between perceived need and help-seeking	Include specific items assessing whether help-seeking followed perceived need.	Example item: “When I needed psychosocial support for my stress, I sought it” (Yes/No)
3. Detailed specification of support sources	Report the support source at a granular level, not just as “formal” or “informal.”	E.g., Occupational health services, general practitioners, psychologists, line managers, peers, friends, family members
4. Exact wording of assessment items	Provide the full, original wording of all items used to assess perceived need, help-seeking behaviour, and support sources.	Enhances transparency and enables accurate synthesis and comparison across studies.
5. Psychological distress / Mental health history	Report whether participants had prior or current psychological distress or mental health problems, and specify how this was measured.	E.g., self-reported diagnosis, clinical screening tools, or symptom inventories.
6. Demographic and background data	Collect and report gender, age, profession, and career stage. Conduct intersectional analysis where possible.	Improves contextual interpretation and enables subgroup comparisons across diverse professional and demographic groups.

## Conclusion

Research on the mental health and well-being of health and social care professionals has increased in recent years, particularly in response to the COVID-19 pandemic. However, to our knowledge, this is the first systematic review specifically investigating the perceived need for psychosocial support and help-seeking behavior among this workforce.

This study highlights a high level of perceived need for support among health and social care professionals. Identifying and addressing this need is essential, not only for improving the mental health of professionals but also for ensuring the continuity of health and social care services, which ultimately impacts patient safety.

Another key finding is the significant discrepancy between the perceived need for and the actual seeking of support. Professionals are more likely to seek help from informal sources rather than from trained therapists or mental health professionals. Understanding both the gap between perceived need and help-seeking, as well as the preferred sources of support, is essential for designing more effective interventions that align with professionals’ needs and preferences. This, in turn, could contribute to the long-term sustainability of the health and social care workforce.

Further research is needed to better understand the relationship between the need for support and help-seeking behavior, as well as the factors influencing these dynamics. Future studies should also focus on evaluating the costs of untreated mental health problems among health and social care professionals, as well as the cost-effectiveness of different interventions designed to prevent and manage these challenges. Future research should incorporate an intersectional analysis of both perceived need for support and help-seeking behaviour. Factors such as gender, ethnicity, professional role, and career stage may significantly influence these outcomes and should be systematically examined. Additionally, institutional culture and power dynamics—particularly within the medical profession—may play a critical role in shaping barriers to help-seeking, especially among doctors. Further conceptual exploration of these structural and cultural influences would enhance our understanding of why support is not sought. To facilitate meaningful synthesis and comparison, future studies should report findings on perceived need and help-seeking in a standardized and transparent manner. Finally, comparative studies examining help-seeking behaviours across different occupational or general populations would provide important contextual insights.

## Supplementary Information

Below is the link to the electronic supplementary material.

Supplementary Material 1

## Data Availability

The datasets used and/or analysed during the current study are available from the corresponding author on reasonable request.
